# Hepatocyte-Derived Lipotoxic Extracellular Vesicle Sphingosine 1-Phosphate Induces Macrophage Chemotaxis

**DOI:** 10.3389/fimmu.2018.02980

**Published:** 2018-12-19

**Authors:** Chieh-Yu Liao, Myeong Jun Song, Yandong Gao, Amy S. Mauer, Alexander Revzin, Harmeet Malhi

**Affiliations:** ^1^Division of Gastroenterology and Hepatology, Mayo Clinic, Rochester, MN, United States; ^2^Department of Physiology and Biomedical Engineering, Mayo Clinic, Rochester, MN, United States

**Keywords:** non-alcoholic steatohepatitis, palmitic acid, sphingosine kinase, sphingolipid signaling, ceramide

## Abstract

**Background:** The pathophysiology of non-alcoholic steatohepatitis involves hepatocyte lipotoxicity due to excess saturated free fatty acids and concomitant proinflammatory macrophage effector responses. These include the infiltration of macrophages into hepatic cords in response to incompletely understood stimuli. Stressed hepatocytes release an increased number of extracellular vesicles (EVs), which are known to participate in intercellular signaling and coordination of the behavior of immune cell populations via their cargo. We hypothesized that hepatocyte-derived lipotoxic EVs that are enriched in sphingosine 1-phosphate (S1P) are effectors of macrophage infiltration in the hepatic microenvironment.

**Methods:** Lipotoxic EVs were isolated from palmitate treated immortalized mouse hepatocytes and characterized by nanoparticle tracking analysis. Lipotoxic EV sphingolipids were quantified using tandem mass spectrometry. Wildtype and S1P_1_ receptor knockout bone marrow-derived macrophages were exposed to lipotoxic EV gradients in a microfluidic gradient generator. Macrophage migration toward EV gradients was captured by time-lapse microscopy and analyzed to determine directional migration. Fluorescence-activated cell sorting along with quantitative PCR and immunohistochemistry were utilized to characterize the cell surface expression of S1P_1_ receptor on intrahepatic leukocytes and hepatic expression of S1P_1_ receptor, respectively.

**Results:** Palmitate treatment induced the release of EVs. These EVs were enriched in S1P. Palmitate-induced S1P enriched EVs were chemoattractive to macrophages. EV S1P enrichment depended on the activity of sphingosine kinases 1 and 2, such that, pharmacological inhibition of sphingosine kinases 1 and 2 resulted in a significant reduction in EV S1P cargo without affecting the number of EVs released. When exposed to EVs derived from cells treated with palmitate in the presence of a pharmacologic inhibitor of sphingosine kinases 1 and 2, macrophages displayed diminished chemotactic behavior. To determine receptor-ligand specificity, we tested the migration responses of macrophages genetically deleted in the S1P_1_ receptor toward lipotoxic EVs. S1P_1_ receptor knockout macrophages displayed a marked reduction in their chemotactic responses toward lipotoxic palmitate-induced EVs.

**Conclusions:**Palmitate-induced lipotoxic EVs are enriched in S1P through sphingosine kinases 1 and 2. S1P-enriched EVs activate persistent and directional macrophage chemotaxis mediated by the S1P_1_ receptor, a potential signaling axis for macrophage infiltration during hepatic lipotoxicity, and a potential therapeutic target for non-alcoholic steatohepatitis.

## Introduction

Non-alcoholic fatty liver disease (NAFLD), the most frequent chronic liver disease worldwide, is characterized by hepatic steatosis (1). The vast majority of NAFLD patients have hepatic steatosis alone, termed non-alcoholic fatty liver (NAFL). Up to 20% of NAFLD patients develop non-alcoholic steatohepatitis (NASH) characterized by hepatocellular injury including ballooning, inflammation and the potential to develop hepatic fibrosis. The underlying importance of inflammation in NASH progression is well-recognized. In natural history studies with paired liver biopsies over time, it was noted that inflammation was the best predictor of future fibrosis risk (2). Mechanistically, preclinical and human data suggest that hepatocyte lipotoxicity, caused by the presence of excessive saturated free fatty acids (FFAs) and other toxic lipid moieties, is involved in provoking or perpetuating inflammation in NASH (3). While pathologic hepatocyte lipoapoptosis has been shown to contribute to proinflammatory responses in the liver (4), recent studies have focused on signaling events arising from damaged or stressed cells before the onset of cell death, such as extracellular vesicles (EVs) in mediating proinflammatory responses in the injured liver (5, 6).

Two notable features of inflammation in NASH are an increase in infiltrating immune cells and an increase in proinflammatory activation of immune cells (7). Enhanced lobular inflammation due to accumulation of a mixed inflammatory infiltrate that includes proinflammatory macrophages within hepatocyte cords is a diagnostic histologic feature of NASH (8). Many patterns of macrophage accumulation are recognized in NASH including clusters termed microgranulomas, fat droplet containing lipogranulomas, and surrounding ballooned hepatocytes termed “satellitosis” (8). Other cell types including lymphocytes, eosinophils, and neutrophils have also been observed in lobular inflammation. More recent studies have implicated other cells of the innate and adaptive immune system in portal inflammation (CD68, CD3, CD8, CD4, CD20, and neutrophil elastase) (9). The links between lipotoxic hepatocytes and immune cells, specifically, hepatocyte-originating signals that direct inflammatory cell infiltration, are not well-defined. Previous studies have defined the role of soluble mediators, such as the chemokine C-C motif chemokine ligand 2/monocyte chemoattractant protein 1 (CCL2/MCP1) and the receptors C-C motif chemokine receptor (CCR) 2 and 5 in NASH (10). In experimental mouse NASH, pharmacologic inhibition of CCR2 and 5 diminishes the recruitment of proinflammatory macrophages leading to amelioration of liver injury and inflammation (11). In a phase 2b human NASH trial of a dual CCR2/5 inhibitor, improvements in liver histology and fibrosis were reported (12). However, complete resolution of macrophage accumulation and inflammation has not been reported highlighting the need to understand additional macrophage recruitment signals. EVs are important cell-derived mediators of intercellular communication (6, 13, 14). These nanometer-sized vesicles participate in short and long-range signaling under normal and stressed conditions (13, 15, 16). The ability of macrophages to migrate through tissues to sites of injury is fundamental to their function as a part of the host innate immunity and inflammation (17). Whether hepatocyte-derived lipotoxic EVs contribute to macrophage infiltration in the hepatic microenvironment and the signaling mechanisms by which macrophages are recruited under lipotoxic stress conditions, still remains incompletely understood.

Palmitic acid (PA), the most abundant saturated FFA found in humans, which is elevated further in NASH, is a well-defined lipotoxic stressor (18). Hepatocytes treated with PA release an increased number of EVs (6, 19), which is in accordance with current evidence suggesting that circulating EVs are increased in NASH and correlate with macrophage accumulation in the liver (5, 20, 21). We have previously demonstrated that PA induces the release of ceramide-enriched EVs via the *do novo* synthesis of ceramide in the endoplasmic reticulum. Further, these EVs contain elevated levels of sphingosine 1-phosphate (S1P), which others have demonstrated to be important for the formation of a subtype of EVs known as exosomes (5, 22). S1P is a potent, bioactive sphingolipid known to regulate immune cell trafficking; and implicated in liver inflammation in NASH (23–25). However, the role of EV S1P cargo in macrophage recruitment is not well-understood. Formation of S1P occurs through the phosphorylation of sphingosine, a ceramide derivative, by sphingosine kinases (SphK) 1 and 2 (24, 26, 27). Our objective was to determine whether SphK-mediated production of S1P in EVs promotes macrophage recruitment via stimulation of the S1P_1_ receptor. We demonstrate that SphK1 and SphK2 mediate the formation of S1P in PA-induced EVs, such that resulting EVs are enriched in S1P cargo to stimulate macrophage migration. Furthermore, macrophages express the S1P_1_ receptor needed for stimulation through the S1P signaling axis. When SphK1 and 2 are inhibited, EV S1P cargo is attenuated and macrophage chemotaxis toward these EVs is reduced. Thus, S1P is an important effector of macrophage chemotaxis, and likely important in this phenomenon *in vivo*. In support of this, the hepatic expression of S1P_1_ receptor is increased in a murine dietary model of NASH. Our observations provide a potential mechanism for the observed macrophage infiltration in NASH, and identify a potential novel therapeutic target.

## Materials and Methods

### Bone Marrow-Derived Macrophages (BMDM)

This study was carried out in accordance with the recommendations of the public health policy on the humane use and care of laboratory animals. The protocol was approved by the Institutional Care and Animal Use Committee (IACUC) of the Mayo Clinic. Bone marrow-derived macrophages (BMDM) were isolated from hind legs of wild type C57BL/6 mice as previously described by us (28). Once euthanized, the mouse was sprayed with 70% ethanol and the skin was cut open using sterile scissors to expose the hind legs. Cuts were made through the hip and ankle joints to remove each leg. The legs were placed in 70% ethanol for 2–3 min, then placed in phosphate-buffered saline (PBS) on ice. In the tissue culture hood, the leg muscle and epiphyses were removed and bone marrow flushed out onto a petri dish using a syringe and 25 gauge needle containing BMDM media. The BMDM media used for bone marrow differentiation consists of Roswell Park Memorial Institute (RPMI)-1640 supplemented with 20% L929 cell-conditioned medium (LCM), 10% fetal bovine serum (FBS), penicillin (100 units/mL) and streptomycin (100 μg/mL). The flushed media containing bone marrow was drawn through a 23 gauge needle 4–5 times to remove clumps. Bone marrow cells were plated onto 150 mm petri dishes (BD Falcon, Oxford, UK) and incubated at 37°C, 5% CO_2_. BMDM media was changed every 2 days on Day 3 and Day 5, and BMDMs were dissociated with Accutase and used in experiments on Day 7.

### Immunohistochemistry for S1P_1_ Receptor

Diet-induced obesity and insulin resistance with concomitant NASH in mice fed a diet high in fat, fructose and cholesterol (FFC) has been well-characterized by us (29, 30). We utilized available archived formalin fixed paraffin embedded liver tissue samples from a previously published, IACUC approved, study where male mice had been fed the FFC diet for 20 weeks for S1P_1_ receptor immunohistochemistry (30). Diet-induced NASH, liver injury and macrophage-mediated inflammation in this cohort have been previously published. Five μM liver sections were dewaxed and rehydrated through graded alcohols. Antigen retrieval was performed by heating in Tris-EDTA buffer (10 mM Tris 1 mM EDTA, pH 9.0) for 10 min at 95°C. After blocking endogenous peroxidase with 3% H_2_O_2_ in distilled water for 10 min, the sections were incubated in blocking buffer provided in the Vectastain ABC staining kit (Vector Laboratories), followed by anti-S1P_1_ receptor antibody (1:100 dilution, Product # 55133-1-1AP, Proteintech) at 4°C in a humidified chamber, overnight. After washing in PBS, slides were incubated with biotin-conjugated secondary antibody, streptavidin conjugated to horseradish peroxidase, and lastly chromogenic substrate per the manufactures instructions (ABC, Vector Laboratories). Sections were dehydrated through graded alcohols and mounted using Permount mounting media (Sigma). Microscopy was performed using the Nikon NIS-Elements advanced research imaging software attached to a Nikon Eclipse TE300 microscope (Nikon, Japan), and the positive areas were captured at 20 × magnification with uniform settings of magnification, light, and exposure time for quantitative image analysis.

### S1P_1_ Receptor Knockout Bone Marrow-Derived Macrophages

Myeloid cell specific knockout mice (KO) mice were generated by crossing mice that express Cre recombinase under the lysozyme 2 promoter (lyzMcre) with mice having the S1P_1_ exon 2 flanked by loxP recombination sites (The Jackson Laboratory Stock # 019141). The S1P${}_{1}^{\text{loxp}}$ mice were generated by Richard Prolia and have been previously described (31). KOs were confirmed by genotyping and quantitative real time PCR (qPCR). BMDM were generated as described above.

### Cell Culture

Immortalized mouse hepatocyte (IMH) cell-line has been previously described by us (5). Cells were cultured in Dulbecco's Modified Eagles Media (DMEM, Life Technologies) supplemented with 10% fetal bovine serum (FBS), glucose (4.5 g/L), penicillin (100 units/mL), and streptomycin (100 μg/mL).

### Extracellular Vesicle Isolation

IMH cells were grown to 90% confluency in 150 mm tissue culture treated dishes. Cells were washed with PBS twice and treated with treatment media containing 400 μM palmitic acid (PA), 400 μM PA + 2 μM of MP-A08 inhibitor, or vehicle control for 20 h. Treatment media consisted of DMEM supplemented with 5% FBS (exosome-free), prepared by overnight ultracentrifugation at 100,000 × g for 16 h, and 1% bovine serum albumin (fatty acid free). Following the 20-h treatment, the cell culture supernatant was collected and centrifuged at 2,000 × g for 20 min to remove cells and cellular debris. Subsequently, the supernatant was extracted and ultracentrifuged at 10,000 × g for 40 min. Supernatant was again removed and further ultracentrifuged at 100,000 × g for 90 min to pellet EVs. The EV pellet was re-suspended in PBS and spun at 100,000 × g for 90 min again to wash the EVs. Pellets were re-suspended in PBS and characterized by nanoparticle tracking analysis or stored at −80°C until further use.

### Caspase 3/7 Assay

IMH cells were plated at a density of 80,000/cm^2^, attached for 24 h, and treated as above for 20 h. Following treatment, caspase 3/7 activity was measured using the Apo-ONE homogeneous caspase-3/7 assay (Promega) according to the manufacturer's instructions and previously described by us (32).

### Morphologic Assessment of Apoptosis

IMH cells were plated in 6 well plates at a density of 80,000/cm^2^, attached for 24 h and treated as above. Nuclear staining with 4′,6-diamidino-2-phenylindole (DAPI) and fluorescence microscopy were used to count total and apoptotic cells; at least 200 cells were counted per condition. Nuclear changes of chromatin condensation and nuclear fragmentation were considered apoptotic (32).

### Sphingosine Kinase Activity

The activity of sphingosine kinases 1 and 2 in cells treated with palmitate with and without the pharmacologic inhibitor, MP-A08, was measured by utilizing a commercially available sphingosine kinase activity assay (Echelon Biosciences). Briefly, IMH cells were plated and treated as described above. Cell lysates were prepared in the provided buffer by freeze thaw followed by sonication. The assay was set up per manufacturer instructions and luminescence normalized to protein content.

### Nanoparticle Tracking Analysis

The Nanosight NTA NS300 (Malvern Instruments, UK) was used to characterize EVs through the analysis of light scatter and Brownian motion of each sample (33). EV samples were diluted with PBS accordingly to maintain a concentration of 2E+08 to 8E+08 particles/mL, and a frame rate in between 10 and 40 particles/frame. Each sample was pumped through the observation chamber by a syringe pump at a constant rate of 25 μL/min. For each sample, 3 videos of 30 s were captured and analyzed by the nanoparticle tracking (NTA) software to obtain the concentration of particles (particles/mL) and size (nanometers). The EV concentration is normalized to the number of cells.

### Lipidomics

EV sphingolipids were quantified by tandem mass spectrometry at the Mayo Clinic Metabolomics Core Laboratory, as described previously (34). EVs isolated from equal number of cells treated with vehicle, PA, or PA plus MP-A08 were analyzed to quantify changes in sphingolipid content.

### CRISPR/Cas9 Gene Editing

SphK2 and SphK1 knockout cell lines for IMH cells were created using the Guide-it CRISPR/Cas9 system (TaKaRa Biotechnology Inc. Japan). The online tool (http://crispr.mit.edu) was used to determine the sgRNA target sequence for mouse SphK2 (5′-TGCGTGCACGCTGCGTCGTCCGG-3′) and mouse SphK1 (5′-ATATATTGCAGTGACGCGTG-3′). Oligonucleotides were annealed and cloned into the pGuide-it Vector (Clontech, Palo. Alto, CA), plasmids isolated, then transfected onto HEK293T cells using Lenti-X Packaging Single Shots (VSV-G) following manufacturer's instructions (Clontech). Virus was harvested at 48 h and filtered through a 0.45 μM pore cellulose acetate filter (Millipore). Virus potency was verified by using Lenti-X GoStix (Clontech), then transferred onto wild type IMH cells for infection. Individual infected cells were flow-sorted into 96-well plates by green and red fluorescence for SphK2 and SphK1, respectively.

### RNA Purification, Reverse Transcription-PCR, and Quantitative Real Time PCR

IMH or BMDM cells were washed with PBS, centrifuged, and pellets were suspended in Trizol. Flash-frozen cryo-preserved liver tissue from our previously published study described above was homogenized in Trizol (30). Total RNA was extracted using the Quick-RNA MiniPrep kit (Zymo Research. Irvine, CA), and the RNA yield was assessed by NanoDrop ND1000 (ThermoScientific, Waltham, MA). Reverse transcription of RNA into cDNA was performed with the iScript cDNA synthesis kit (Bio-Rad Laboratories, Hercules, CA). PCR amplification was run using the EmeraldAmp MAX Master Mix (TaKaRa) on the Tetrad Thermal Cycler (MJ Research Inc. Watertown, MA). Amplified products were analyzed by QIAxcel Advanced System (Qiagen). Quantitative real time PCR was carried out using the LightCycler 480 SYBR Green I Master Mix (Roche), and run on LightCycler 480 (Roche). Primers used for mouse SphK2 include: forward 5′-TTTACGAGGTGCTGAATGGG-3′ and reverse 5′-ACCGACAACCTGCTCAAAC-3′. Primers for mouse SphK1 include: forward 5′-TGAATGGGCTAATGGAACGG-3′ and reverse 5′-GTCTTCATTAGTCACCTGCTCG-3′. HPRT was used as the house keeping gene for qPCR using primers: forward 5′- TCAGTCAACGGGGGACATAAA-3′ and reverse 5′- GGGGCTGTACTGCTTAACCAG-3′. For S1P_1_ receptor 5′-ATGGTGTCCACTAGCATCCC-3′ and 5′-CGATGTTCAACTTGCCTGTGTAG-3′ primers were utilized for both qPCR and standard reverse transcription PCR. For standard reverse transcription PCR primers for mouse S1P_2_: forward 5′-ATGGGCGGCTTATACTCAGAG-3′ and reverse 5′-GCGCAGCACAAGATGATGAT-3′; S1P_3_: forward 5′-ACTCTCCGGGAACATTACGAT-3′ and reverse 5′-CAAGACGATGAAGCTACAGGTG-3′; S1P_4_: forward 5′-GTCAGGGACTCGTACCTTCCA-3′ and reverse 5′-GATGCAGCCATACACACGG-3′; S1P_5_: forward 5′-GCTTTGGTTTGCGCGTGAG-3′ and reverse 5′-GGCGTCCTAAGCAGTTCCAG-3′; and 18S: forward 5′-CGCTTCCTTACCTGGTTGAT-3′ and reverse 5′-GAGCGACCAAAGGAACCATA-3′. PCR products were resolved by capillary electrophoresis using the QIAxcel Advanced System (Qiagen).

### Microfluidic 2D Cell Migration Chamber

The microfluidic gradient generator is a 2D cell migration chamber that allows for a passive generation of a stable gradient in space and time. The device itself consists of a piece of PDMS bonded to a glass coverslip, and a network of micro-channels embedded in between (Supplementary Figure 1). The microfluidic devices were fabricated using standard soft lithography approaches. The design of the gradient generator was adapted from previous reports (35). The two circular wells on top are inlets for the chemoattractant (RPMI-1640 + EVs) and chemoattractant-free media (RPMI-1640). Immediately after the inlets is a sequence of balance and equilibrium channels, then a network of microchannels that combines and mixes the chemoattractant as they flow through. Each resulting channel at the end of the network contains a different proportion of the chemoattractant such that the gradient will be established in the cell chamber perpendicular to the direction of flow (34). BMDMs were dissociated with Accutase on Day 7 and reconstituted in complete BMDM media to give a concentration of 1E6 cells/mL. The cells were then seeded into the cell chamber of the gradient generator by backflow through the bottom port. Once the cells were spread throughout the chamber, the device was incubated at 37°C, 5% CO_2_ for 1 h to allow the cells to adhere. Subsequently, 200 μL of serum free RPMI-1640 or serum free RPMI-1640+EVs was added to the left and right inlets at the top, respectively. The device was allowed to sit for 10 min for the media to flow through the micro-channels before image capture was begun.

### Time-Lapse Microscopy

Following cell seeding and generation of the EV gradient, the microfluidic gradient generator was placed in the stage incubator. Images of macrophage migration in the cell chamber were captured every 15 s for 4 h using the 5x objective, Definite Focus (Zeiss) and the ZEN 2.3 lite software (Zeiss).

### Cell Tracking and Analysis

All time-lapse images acquired through the Zen Pro imaging software (Zeiss) were exported as time-lapse videos (100 frames/second). The resulting video file was imported into ImageJ (National Institutes of Health, Bethesda, USA), and cells were tracked using the Manual Tracking plugin (Fabrice Cordelières, Institut Curie, Orsay, France). The resulting cell tracks were analyzed using the Chemotaxis and Migration Tool 2.0 (Ibidi GmbH, Munich, Germany). In each experiment, random migrating cells within frame were manually tracked (10–50 cells). The software was also used to plot cell tracks and calculate measured values, including the migratory persistence (forward migration index), angle of migration relative to the gradient axis, velocity, Euclidean and accumulated distances.

### Fluorescence-Activated Cell Sorting (FACS)

Mouse liver was dissociated with the liver dissociation kit, mouse (Miltenyi Biotec, Germany), using the gentleMACS Dissociator (Miltenyi Biotec) following manufacturer's protocols. The liver digest was passed through a 40 μm filter (Falcon 352340; BD Biosciences) to remove large debris and cell clusters. The filtered dissociate was centrifuged at 300 × g for 5 min. The cells were re-suspended in 7 ml 25% Percoll in a 15 ml falcon tube, and 2 ml of 50 % Percoll was slowly laid on the bottom of the same tube with a glass Pasteur pipet. The tube was spun at 2,000 rpm, brakes removed, for 20 min at 4°C. After the tube was carefully removed from the centrifuge, 2–3 ml of debris was removed from the top of the gradient. Using a glass Pasteur pipet, cells from the band in between the middle colored interface and transparent interface below were removed without disturbing the RBC pellet at the bottom. To dilute out the Percoll, cells were transferred to a 50 ml falcon tube, the volume was brought up to 40 ml with SF-RPMI, and spun at 400 × g for 10 min. The supernatant was removed and cells re-suspended in PEB buffer, containing PBS, 0.5% bovine serum albumin (BSA) and 2 mM EDTA. Viable cells were counted on a hemocytometer using Trypan Blue (Gibco). Cells were then incubated with FcR Blocking Reagent (Miltenyi Biotec) for 5 min at 4°C, followed by CD45 conjugated to VioBlue (Miltenyi Biotec), F4/80 conjugated to APC-Cy7 (Biolegend), and mouse S1P_1_-phycoerythrin (PE) conjugated antibody (RandD Systems) for 10 min at room temperature in the dark. Cells were centrifuged at 400 × g for 6 min at room temperature. Cells were washed once with PEB buffer, and centrifuged for 5 min at 400 × g. The supernatant was removed and cells were re-suspended in 1% paraformaldehyde. Flow cytometry was performed with the MACSQuant Analyzer 10 (Miltenyi Biotec). The stained population was acquired by gating side scattered light and forward scattered light to exclude the dead cell population, gated on CD45 positive and fluorescence minus one controls were used to identify and gate the cell population expressing both F4/80 and S1P_1_ receptor. F4/80 and S1P_1_ receptor expressing population is expressed as a percentage of CD45 high intrahepatic leukocytes. Graphs were made in Flowlogic software (Miltenyi Biotec).

### Statistical Analysis

Statistical analysis and graphs were made in GraphPad Prism version 6.00 for Windows (GraphPad Software, La Jolla, CA). Mean ± S.E.M. are presented in each graph. The two-tailed student's *t*-test was used to compare groups, and the results were statistically significant when the *p*-value was < 0.05. The Rayleigh Test of Uniformity was used to test a circular distribution of cell end points, and was performed using the Chemotaxis and Migration Tool 2.0. Rejection (*p* < 0.05) of the null hypothesis, which states that the distribution of cell end points is uniform, showed that there is sample mean direction of migration.

## Results

### Macrophages Migrate Directionally and Persistently Toward PA-Induced EVs

**Figure 1:**
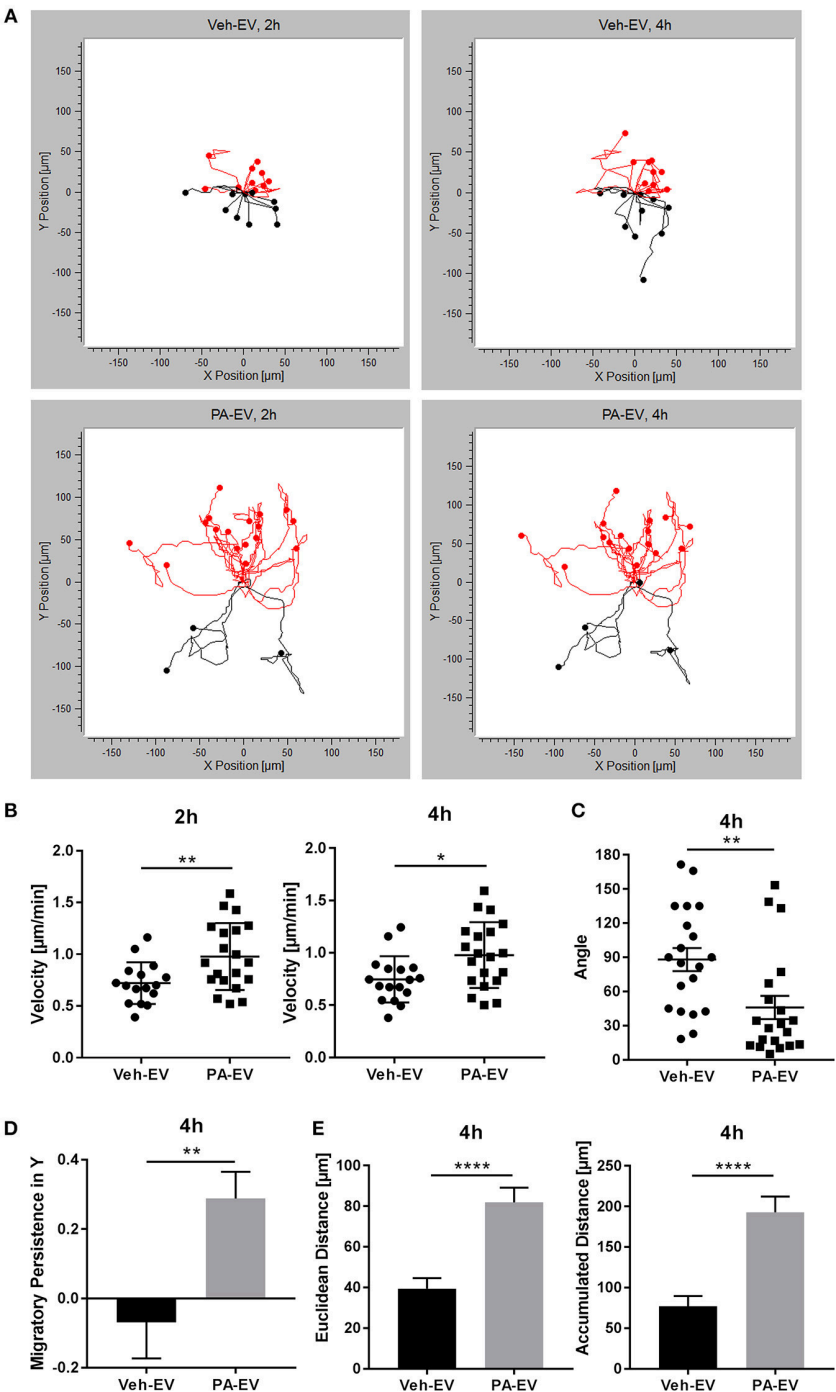
PA-EVs are chemoattractive to macrophages. **(A)** Representative migration plots for 2D chemotaxis assay, showing BMDM migration trajectories of 20 randomly selected cells at 2 and 4 h (*n* = 3). BMDMs were seeded in the microfluidic gradient generator for 1 h, exposed to a Veh-EV or PA-EV gradient for 4 h, and captured by time-lapse microscopy. EVs were isolated from the same number of IMH cells treated with 400 μM PA or vehicle for 20 h. Cells highlighted in red are migrating up toward the positive end of the gradient. The Rayleigh test for uniformity was used to test whether the cell distribution was uniform. **(B)** Migration velocity when BMDMs were exposed to a Veh-EV vs. PA-EV gradient at 2 and 4 h (*n* = 3). **(C)** Angle between the cell's leading edge and gradient axis at 4 h in a Veh-EV vs. PA-EV gradient (*n* = 3). A more acute angle indicates that migration was more consistent with the gradient axis. **(D)** Migratory persistence in Y of BMDMs treated with Veh-EV vs. PA-EV gradient at 4 h (*n* = 3). Strong chemotaxis effects are characterized by higher migratory persistence in Y. **(E)** Euclidean distance (shortest distance between the start and end points) and accumulated distance (total path length) between BMDMs migrating in a Veh-EV or PA-EV gradient for 4 h (*n* = 3). ^*^p < 0.05, ^**^p < 0.01, ^****^p < 0.0001. All error bars are SEM.

Hepatic lipotoxicity, caused by the presence of excessive saturated free fatty acids (FFAs), and its related macrophage recruitment is implicated in NASH pathogenesis (19, 36). Previously, we found that PA promotes stress-induced release of EVs through the ER stress sensor IRE1α (5). To understand recipient cell responses, we asked whether PA-induced lipotoxic EVs could be a mediator of macrophage recruitment into the liver. Proinflammatory intrahepatic macrophages in NASH are derived from bone marrow-derived monocytes; therefore, we used BMDMs in these experiments as a surrogate for monocyte-derived macrophages. BMDMs were exposed to a Veh-EV or PA-EV gradient (EVs isolated from the same number of hepatocytes treated with vehicle or PA) through the microfluidic gradient generator for 4 h. BMDMs migrated directionally (Rayleigh Test *P* < 0.05) when exposed to PA-EVs but not when exposed to Veh-EVs (Figure 1A). BMDMs migrated at a higher velocity when exposed to PA-EVs throughout the duration of exposure, and at more acute angles toward PA-EVs (Figures 1B,C). A positive migratory persistence in Y [sum of the ratio of displacement along the gradient axis (Y axis) to total accumulated path length of all cell tracks, averaged] indicated that the direction of macrophage migration was toward the positive end of the PA-EV gradient (Figure 1D), and they did so over greater distances (Figure 1E). Together, these measured values show that macrophages migrate faster, longer, and in a more directionally persistent manner toward PA-EVs.

### Inhibition of SphK1 and SphK2 Reduces S1P Levels in PA-Induced EVs

**Figure 2:**
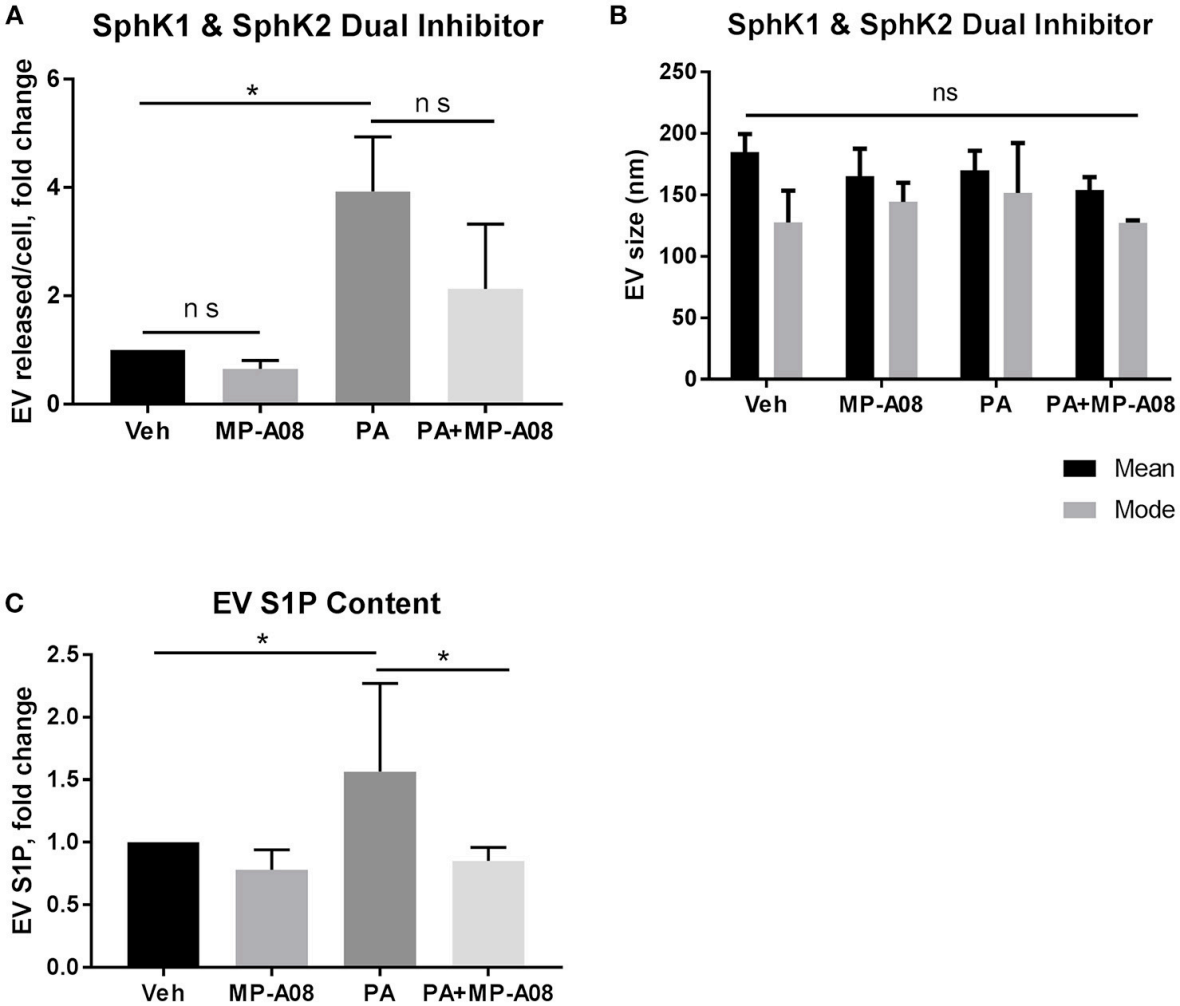
EV S1P is not elevated under SphK1 and SphK2 inhibition. **(A)** IMH cells were treated with 400 μM PA, 400 μM PA + 2 μM MP-A08, vehicle, or vehicle + 2 μM MP-A08 for 20 h. EVs released were isolated by differential ultracentrifugation and characterized by NTA and normalized to cell numbers. Data are pooled from 4 experiments. **(B)** Size (nm, nanometers) of EVs released by IMH cells treated with vehicle, vehicle + 2 μM MP-A08, 400 μM PA, or 400 μM PA + 2 μM MP-A08 for 20 h. Data are pooled from 4 experiments. **(C)** EV S1P content was quantified by tandem mass spectrometry (*n* = 2). ^*^p < 0.05, ns, not significant. All error bars are SEM.

We next examined the EV S1P content and the significance of its formation in the release of PA-induced EVs. S1P is formed through the phosphorylation of sphingosine by SphK1 and SphK2 (24). We attempted to establish a double SphK1 and SphK2 knockout cell line using CRISPR/Cas9 gene editing technology. However, this was not successfully achieved likely due to lethality in the absence of both SphK isoenzymes, similar to observations in mice (37). It is also known that the downregulation of one SphK results in the compensatory upregulation of the other (38). Henceforth, we used a pharmacologic approach in the subsequent experiments. We asked whether SphK1 and SphK2 mediate the formation of S1P in PA-induced EVs by using the dual SphK pharmacologic inhibitor, MP-A08. We treated hepatocytes with vehicle, vehicle + MP-A08, PA, and PA + MP-A08 for 20 h, isolated the EVs released and normalized to cell number. We confirmed the absence of MP-A08-induced apoptosis by the biochemical and morphologic assessment of apoptosis (Supplementary Figures 2A,B). In hepatocytes treated with MP-A08, PA, or PA + MP-A08, both caspase 3/7 activity and percent apoptotic nuclei were comparable to vehicle treated cells. The inhibitory effect of MP-A08 was confirmed by measuring sphingosine kinase activity (Supplementary Figure 2C). We found almost a 3-fold increase in EV release in PA-treated cells compared to vehicle-treated cells, and no significant difference in EV release when SphK1 and SphK2 were inhibited by MP-A08 (Figure 2A). We did not find any difference in vesicle size (nm) between the groups (Figure 2B). We next quantified and analyzed the EV S1P content by tandem mass spectrometry. We found that PA-EVs were enriched in S1P cargo compared to those released by vehicle-treated, vehicle + MP-A08-treated and PA + MP-A08-treated cells (Figure 2C). Altogether, these data suggest that SphK1 and Sphk2 play a crucial role in the formation of S1P in PA-induced EVs, though the number of EV released is unaffected.

### Macrophages Are Chemotactic Toward PA-Induced EVs Through the S1P Signaling Axis

**Figure 3:**
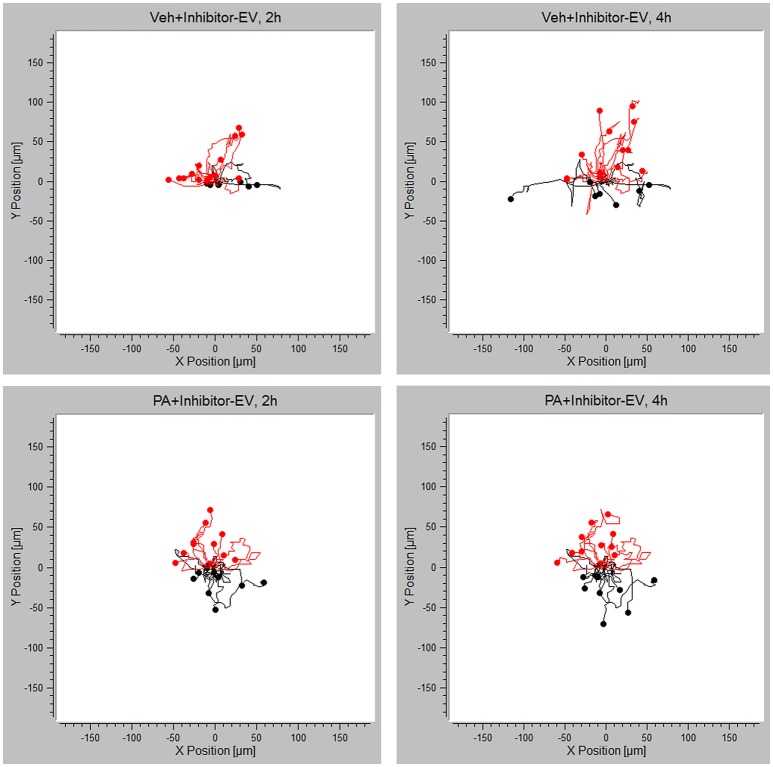
PA-EV chemotactic response is attenuated through inhibition of SphK1 and SphK2. Migration plots of BMDMs migrating in a Veh + MP-A08-EV or PA + MP-A08-EV gradient for 4 h. Plots show migration trajectories of 20 randomly selected cells from each experiment (*n* = 3). The Rayleigh test for uniformity was used to test whether cell distribution was uniform. Cells highlighted in red are migrating up.

**Figure 4:**
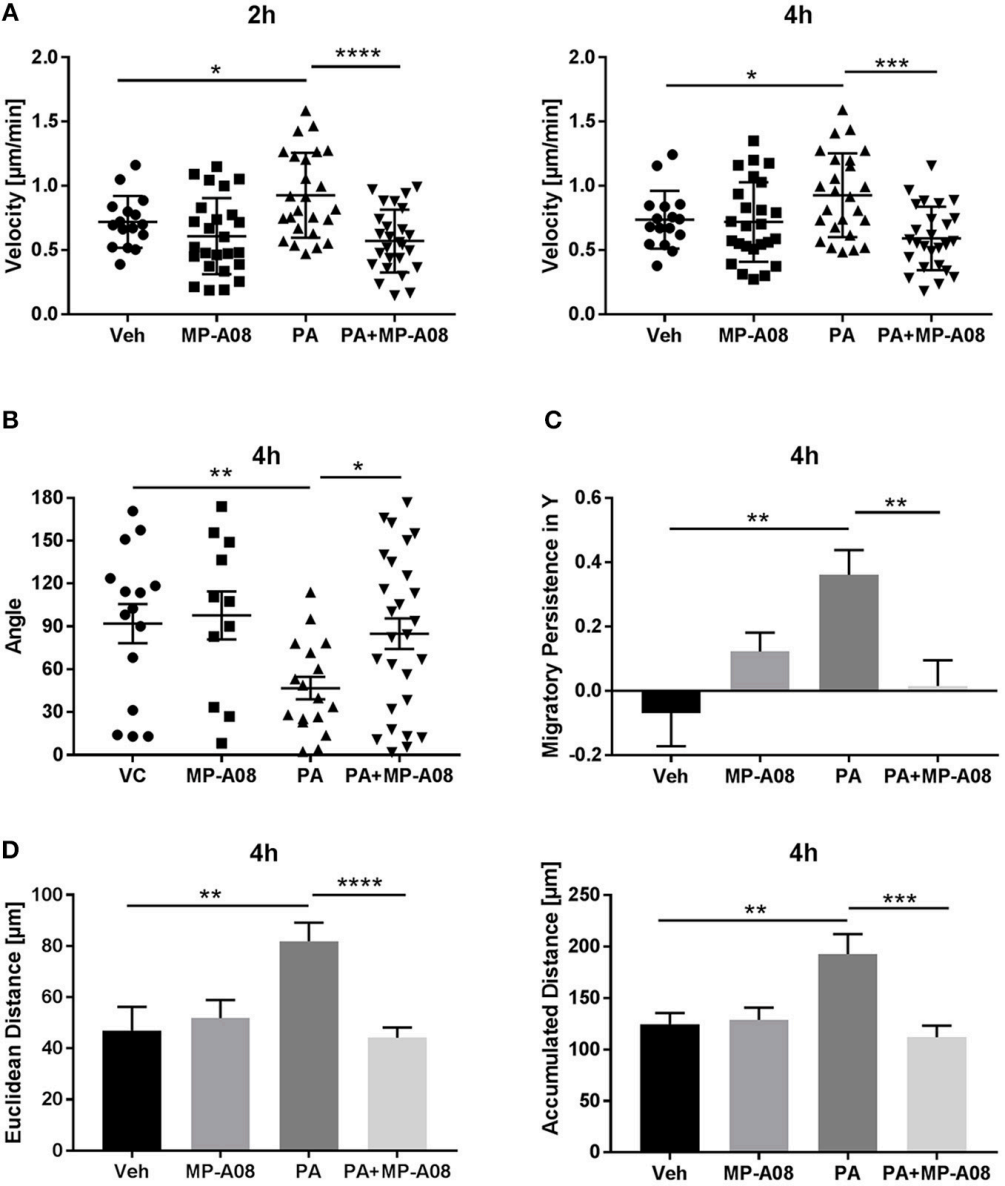
Macrophages migrate persistently and directionally toward S1P-enriched EVs. **(A)** BMDM migration velocity when exposed to Veh-EV, Veh + MP-A08-EV, PA-EV, or PA + MP-A08-EV gradients at 2 and 4 h (*n* = 3). **(B)** The angle between the cell's leading edge and the gradient axis for BMDMs migrating in 4 types of EV gradients for 4 h (*n* = 3). **(C)** Migratory persistence in Y, comparing chemotaxis efficiency of BMDMs migrating in 4 types of EV gradients for 4 h (*n* = 3). **(D)** Comparison of the Euclidean distance and accumulated distance between BMDMs migrating in 4 types of EV gradients for 4 h (*n* = 3). ^*^p < 0.05, ^**^p < 0.01, ^***^p < 0.001, ^****^p < 0.0001. All error bars are SEM.

Having demonstrated that PA-EVs attract macrophages, and that PA-EVs contain elevated levels of S1P through functional SphK1 and SphK2, we next asked whether SphK mediated formation of S1P plays a role in the chemoattractive effects of PA-EVs. If macrophages are stimulated by the SphK mediated formation of S1P cargo in PA-induced EVs, then the inhibition of both SphK1 & SphK2 should inhibit macrophage migration through impairment of the S1P signaling axis. BMDMs were exposed to 4 different gradients of EVs isolated from Veh-treated, Veh + MP-A08-treated, PA-treated, or PA + MP-A08-treated cells for 4 h. BMDMs did not migrate directionally (Rayleigh Test *P* > 0.05) in either the Veh + MP-A08-EV or PA + MP-A08-EV gradient (Figure 3). Migration velocity was significantly reduced toward PA + MP-A08-EVs compared to PA-EVs, similar to the response toward vehicle controls at both 2 and 4 h (Figure 4A). We speculate that this is due to the reduction in S1P content by the SphK inhibitor MP-A08. BMDMs in the PA + MP-A08-EV gradient migrated at less acute angles, or less in alignment with the gradient axis compared to those migrating toward PA-EVs (Figure 4B). Furthermore, the macrophages did not migrate persistently toward the positive end of the PA + MP-A08-EV gradient (Figure 4C), and migration distance was significantly reduced compared to that when exposed to PA-EVs (Figure 4D). Taken together, these data suggest that pharmacologic inhibition of SphK1 and SphK2 and the subsequent reduced levels of S1P in PA-EVs diminish their chemoattractive effects toward macrophages. We speculate that S1P mediated recruitment of macrophages is achieved through stimulating the S1P_1_ receptor on macrophages.

### Intrahepatic Macrophages Express S1P_1_ Receptor

**Figure 5:**
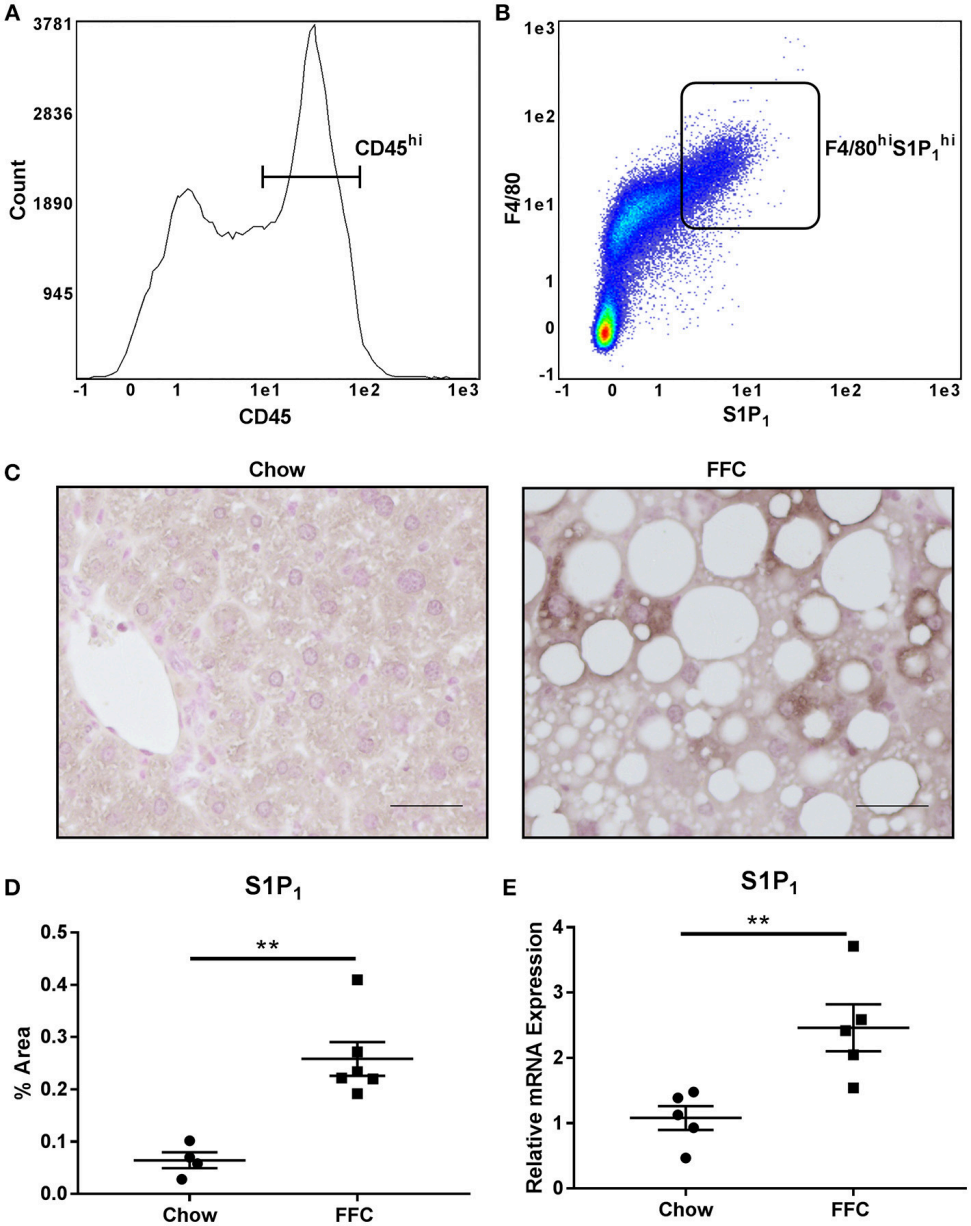
Intrahepatic macrophages express the S1P_1_ receptor. **(A)** CD45 expression and **(B)** F4/80 and S1P_1_ receptor expression of CD45^hi^ intrahepatic macrophages was determined in normal mouse livers by FACS analysis. **(C)** S1P_1_ receptor immunohistochemistry was performed in livers from mice fed a diet high in fat, fructose and cholesterol (FFC) or chow for 20 weeks. Scale bar = 50 μM, *n* = 4 for chow and *n* = 6 for FFC. **(D)** Quantification of immunohistochemistry for S1P_1_ receptor in chow fed (*n* = 4) and FFC fed (*n* = 6) mice. **(E)** mRNA expression for S1P_1_ receptor in chow fed (*n* = 5) and FFC fed (*n* = 5) mice. ^**^p < 0.01.

As S1P_1_ receptor is known to be expressed by macrophages (39), we characterized its expression on intrahepatic macrophages in normal mouse livers through FACS analysis. We defined intrahepatic leukocytes as the CD45^hi^ population and further defined the macrophage population as F4/80^hi^. We found that a significant percentage of the CD45^hi^ population expressed both F4/80 and S1P_1_ receptor (22.5 ± 2% of the CD45^hi^ population, *n* = 4) (Figures 5A,B). Having demonstrated that this receptor is expressed by intrahepatic macrophages, we next asked whether it is upregulated in NASH, which would be in keeping with our proposed model. For this we utilized available archived mouse liver RNA and tissue samples from a previously published study (30). In this study mice were fed a diet known to induce obesity, insulin resistance and NASH, thus recapitulate cardinal features of human NASH, as previously reported by us and others (30, 40). Liver injury and macrophage accumulation in these mouse livers have been previously reported (30). In this dietary NASH model we found a significant increase in the hepatic expression of S1P_1_ receptor by immunohistochemistry (Figures 5C,D), in regions of inflammatory cell infiltration. The mRNA abundance in this dietary NASH model was similar to the protein expression observed by immunohistochemistry (Figure 5E). Altogether, these data support an increase in S1P_1_ receptor expression in the liver in NASH, and are consistent with our model that S1P_1_ receptor expressing macrophages are recruited into the liver in NASH.

### Chemotactic Effect Toward PA-EVs Is Attenuated in the Absence of S1P_1_ Receptor

**Figure 6:**
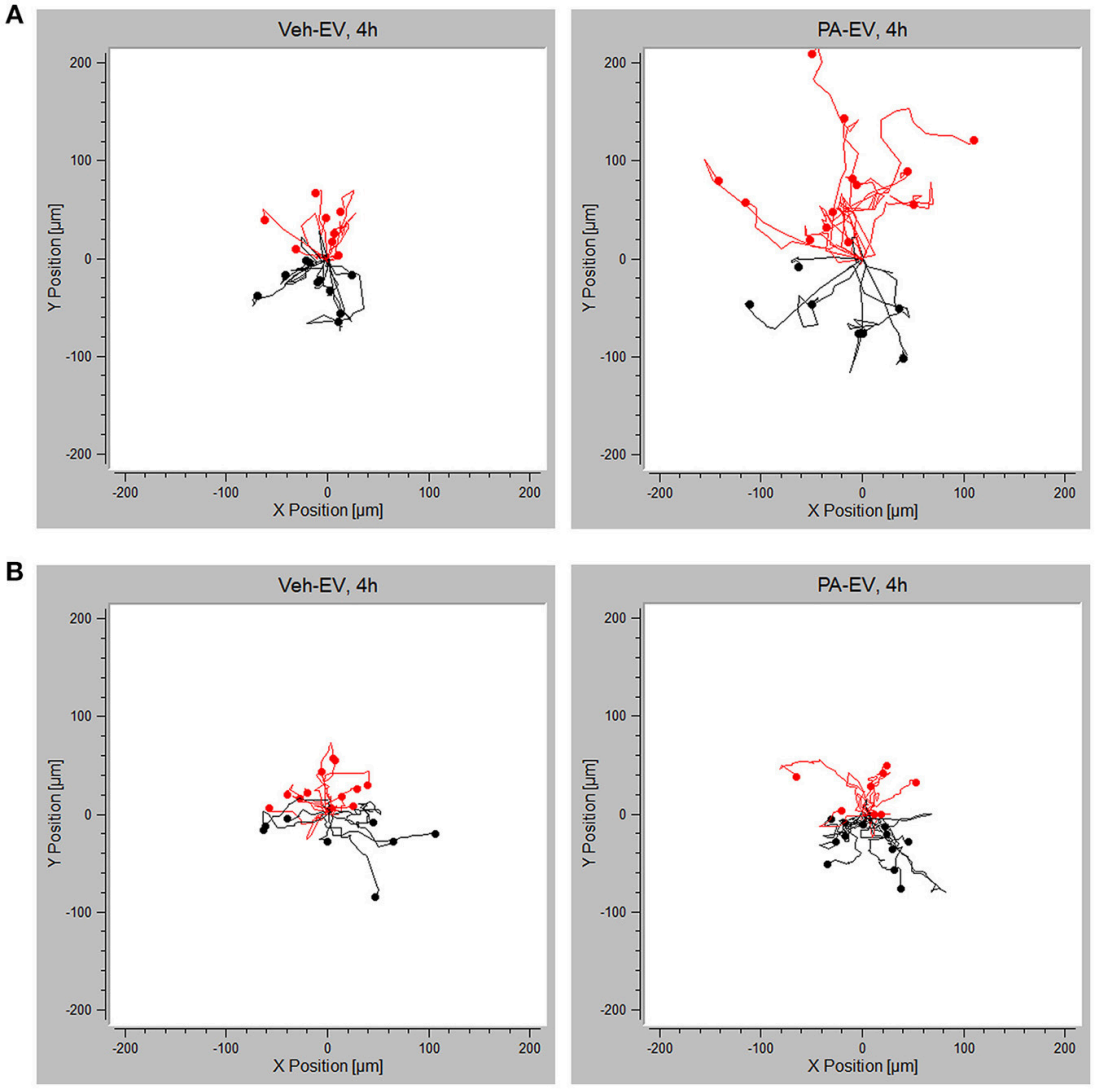
Migration toward PA-EVs is reduced in the absence of the S1P_1_ receptor. **(A)** Migration plots of WT BMDMs migrating in a Veh-EV and PA-EV gradient for 4 h (*n* = 3). **(B)** Migration plots of S1P_1_ receptor KO BMDMs migrating in a Veh-EV and PA-EV gradient for 4 h (*n* = 2). Twenty randomly selected cells from each experiment are shown in each plot. The Rayleigh test of uniformity was used to test whether the cell distribution was uniform. Cells highlighted in red are migrating up toward the positive end of the gradient.

**Figure 7:**
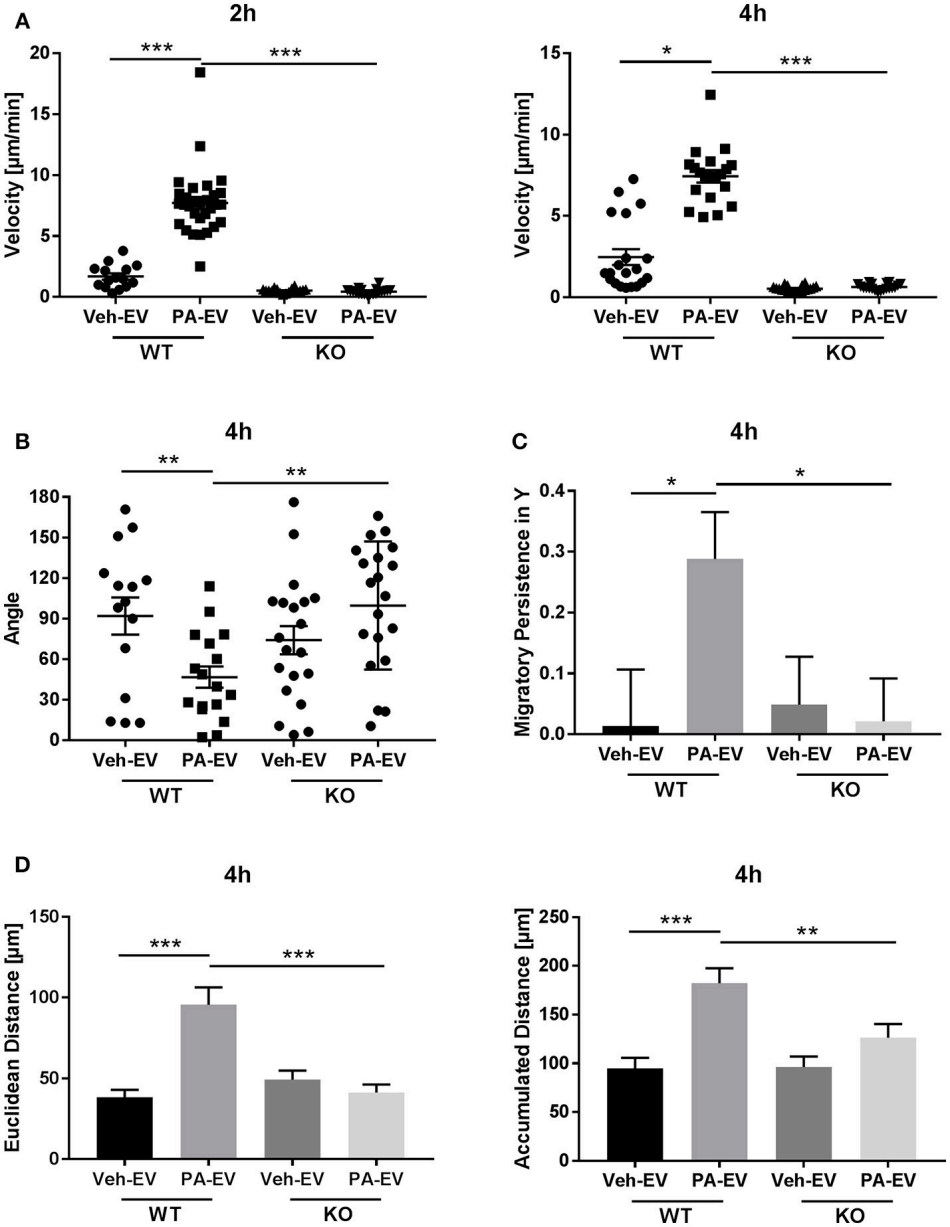
S1P_1_ receptor KO BMDMs migrate at reduced speeds, distances and persistence toward PA-EVs. **(A)** Migration velocity of WT vs. S1P_1_ receptor KO BMDMs in Veh-EV and PA-EV gradients at 2 and 4 h (*n* = 2). **(B)** Angle between the cell's leading edge and gradient axis for WT and S1P_1_ receptor KO BMDMs in Veh-EV and PA-EV gradients for 4 h (*n* = 2). **(C)** Migratory persistence in Y of WT and S1P_1_ receptor KO BMDMs migrating in Veh-EV and PA-EV gradients for 4 h (*n* = 2). **(D)** Euclidean distance and accumulated distance for WT and S1P_1_ receptor KO BMDMs migrating in Veh-EV and PA-EV gradients for 4 h (*n* = 2). ^*^p < 0.05, ^**^p < 0.01, ^***^p < 0.001. All error bars are SEM.

Having demonstrated the presence of S1P_1_ receptor on intrahepatic macrophages, we next wanted to confirm whether S1P_1_ receptor plays a role in macrophage chemotaxis toward S1P in PA-EVs. BMDM S1P_1_ receptor knockout was confirmed by RT-PCR (Supplementary Figure 3); the mRNA expression of S1P receptors 2–5 was unaffected by knockout of S1P_1_ receptor. S1P_1_ receptor KO BMDMs were exposed to Veh-EV or PA-EV gradients in the microfluidic gradient generator for 4 h. S1P_1_ receptor KO BMDMs did not migrate directionally (Rayleigh Test *P* > 0.05) toward PA-EVs compared to WT BMDMs (Rayleigh Test P < 0.05) when exposed to a PA-EV gradient for 4 h (Figures 6A,B). The migration of S1P_1_ receptor KO BMDMs was similar toward Veh-EVs and PA-EVs and comparable to WT BMDMs migration toward Veh-EVs. This suggests that the S1P_1_ receptor is crucial in mediating chemotactic effects in macrophages during S1P signaling. This was further confirmed by measuring migration velocity at 2 and 4 h, showing that S1P_1_ receptor KO BMDMs migrate significantly slower toward PA-EVs compared to WT BMDMs (Figure 7A). KO BMDMs also migrated at less acute angles, with significantly less persistence, and decreased Euclidean and accumulated distances toward PA-EVs (Figures 7B–D). Thus, in the absence of S1P_1_ receptor, all measured parameters show attenuated migration toward PA-EVs, similar to the migration patterns toward MP-A08 treated EVs. This evidence further confirms the involvement of the S1P_1_ receptor in S1P signaling in macrophage chemotaxis.

## Discussion

Macrophage-mediated sterile inflammation is a salient feature of NASH. The signals that lead to the recruitment of proinflammatory monocyte-derived macrophages such that they infiltrate the hepatic parenchyma are not well-understood. In this paper we report that: (i) lipotoxic hepatocyte-derived extracellular vesicles attract macrophages by chemotaxis; (ii) intrahepatic leukocytes express the sphingosine 1-phosphage 1 (S1P_1_) receptor; and (iii) macrophage chemotaxis is mediated by the sphingosine 1-phosphate signaling axis. These *in vitro* findings advance our understanding of how lipotoxic hepatocyte-derived extracellular vesicles are proinflammatory and provide an explanation for the infiltration of macrophages into the hepatic cords by responding to EV gradients in the tissue microenvironment.

Lipotoxicity, summarized as accumulation of toxic lipid species with concomitant tissue or cellular injury, is a fundamental observation in obesity-associated disorders such as NASH (3). Several lipid classes have been implicated in lipotoxicity, including saturated free fatty acids, ceramides, lysophosphatidyl choline and free cholesterol (3, 41, 42). Among these lipids, the saturated free fatty acid palmitate, which is most abundant physiologically, and accumulates further in lipotoxic states is best characterized as being lipotoxic (18). Additionally, lipids, that can be synthesized directly from palmitate, such as ceramide via the *de novo* biosynthesis pathway (43), and indirectly such as lysophosphatidyl choline via phospholipase A2 (44), can induce hepatocyte cell death or stress responses. Though rarely observed in standard H&E stained liver biopsies, lipotoxic hepatocyte cell death correlates with features of progressive NASH (45, 46). However, death is a terminal outcome and there is emerging interest in events that occur in “at-risk” cells prior to the terminal outcome of cell death, which we and others have termed sublethal cellular stress (21). The “at-risk” cells may be defined in many ways including, but not limited to, cells that demonstrate activation of cell stress pathways such as endoplasmic reticulum stress (47), activation of death receptor signaling without cell death (48), secretion of soluble factors, and increasingly, as we demonstrate here, the release of extracellular vesicles (49). EVs are heterogeneous, membrane-enclosed, nanoparticles that most cells secrete into the extracellular space and into circulation (6). EV number and cargo, including lipids, proteins and nucleic acids, changes with disease states and there is immense interest in identifying disease-specific EV signatures, both for their utility as liquid biopsy and as a potentially targetable pathophysiologic mechanism (6, 50, 51). Here we advance the understanding of EV-mediated communication between donor cell, i.e., lipotoxic hepatocytes, and recipient cell, i.e., macrophages.

Proinflammatory activation of the innate immune system, manifest as macrophage infiltration of the hepatic parenchyma, is linked to key features of NASH including hepatocyte injury, hepatocyte cell death and fibrosis (45, 46). While macrophage activation by itself can lead to secretion of proinflammatory cytokines, death ligands and promote fibrosis, in lipotoxic disorders, where hepatocytes are a key cell that accumulates toxic lipids and lipid accumulation precedes the development of overt inflammation, hepatocyte-to-macrophage signals are important in understanding this key signaling axis. Our laboratory has focused on lipotoxic hepatocyte derived EVs as mediators of this cellular cross talk, and in particular signaling lipid mediators on EVs. We have previously demonstrated that palmitate-treated hepatocytes release ceramide-enriched extracellular vesicles, that these vesicles are also enriched in the ceramide-derived signaling sphingolipid S1P, and that their release is dependent on the endoplasmic reticulum stress sensor, inositol requiring protein 1 alpha (IRE1α) (5). Furthermore, we have recently demonstrated that the release of extracellular vesicles in PA-treated hepatocytes is preserved in the presence of caspase inhibitors, highlighting the potential importance of lipotoxic stress-induced EVs in cellular cross talk with the innate immune system (49). Here we extend our earlier observations by demonstrating persistent and directional migration of macrophages toward an EV S1P gradient. Not only are PA-induced EVs enriched in S1P, PA-stimulated cells release more EVs than vehicle-treated cells raising the possibility that macrophage chemotaxis toward S1P may be occurring toward a higher number of EVs. While this is possible, we show a lack of EV-S1P enrichment in cells treated with sphingosine kinase inhibitor MP-A08 and PA without a significant reduction in EV numbers, suggesting that EV cargo, S1P in this case, is mediating the observed macrophage chemotaxis. Others have reported proinflammatory, proangiogenic and profibrotic EVs in NASH models (52–54). Here, we focused on macrophage chemotaxis toward lipotoxic EVs in order to provide an explanation for macrophage infiltration, as chemotaxis to a site of injury and activation are both important components of macrophage effector responses. Though transwell macrophage migration has been reported by us and others in response to lipotoxic EVs, it does not differentiate between chemokinesis and chemotaxis. This is the first report of true directional chemotaxis of macrophages toward a lipotoxic EV gradient in response to EV S1P. We have previously demonstrated that EV S1P is increased in human and murine NASH (5). Thus, lipotoxic PA-EVs may play a role in macrophage recruitment *in vivo* in addition to the previously defined role of chemokines such as CCL2 and chemokine receptors CCR2 and CCR5 (10).

S1P is a potent bioactive sphingolipid, formed by phosphorylation of sphingosine by two isoenzymes of sphingosine kinase, SphK1 and SphK2. The cellular and subcellular distribution of SphK1 and 2 vary, accounting for distinct roles in the compartmental formation of S1P. Sphingosine, in turn, is formed by the deacylation of ceramide. Studies have shown that S1P is involved in the pathogenesis and progression of NASH, with concurrent upregulation of SphK1 and occasionally SphK2 (24). Both ceramide and S1P are implicated in the formation of intraluminal vesicles (ILVs) in multivesicular bodies and in cargo sorting into intraluminal vesicles (5, 22); however, when we inhibited SphK1 and 2 we did not observe a significant reduction in the number of EVs released by hepatocytes; predictably, EV S1P was significantly reduced to levels comparable to basal levels. S1P is the ligand of a family of 5 G-protein couple receptors, S1P_1_-S1P_5_, that regulate downstream signaling to mediate a variety of cellular responses including: immunity, cellular migration, angiogenesis, vascular and cardiac development (55, 56). Of these, S1P_1_ receptor expression and S1P gradients in the microenvironment are known to influence T lymphocyte trafficking (57), NK cell trafficking (25), dendritic cell trafficking (58). Here, we confirmed the cell surface expression of S1P_1_ on intrahepatic macrophages (59) and demonstrate that macrophage chemotaxis is attenuated in response to S1P deficient EVs. Our observations of macrophage chemotaxis toward EV-S1P are consistent with earlier reports of macrophage chemotaxis induced by S1P (60). Further, we have demonstrated that the chemotaxis responses of macrophages genetically deficient in S1P_1_ receptor toward PA-EVs are attenuated, confirming that macrophage chemotaxis toward lipotoxic EVs is mediated by an intact S1P-S1P_1_ signaling axis. Of the 5 known S1P receptors, S1P_1_, S1P_2_, S1P_3_, and S1P_4_ are expressed by human macrophages and S1P_1_, S1P_2_, and S1P_4_ by monocytes (39). Our data suggest that the role of S1P_1_ warrants investigation in human NASH.

Taken together these data provide an *in vitro* mechanism for lipotoxic hepatocyte-derived S1P-enriched EVs in macrophage chemotaxis responses. Recognizing that proinflammatory macrophages are derived from proinflammatory monocytes, we propose a model wherein multiple hepatocyte-derived signals act in concert to mediate macrophage attraction and activation in the microenvironment of NASH livers. Soluble signals such as CCL2 may lead to the egress of proinflammatory monocytes from the bone marrow and their recruitment in to the liver (10, 61); other soluble or EV-mediated signals might home proinflammatory monocytes to the sinusoidal endothelium; and promote adhesion of proinflammatory monocytes to give rise to macrophages which then infiltrate hepatic cords in response to an EV S1P gradient in the local microenvironment. Our data support a key role for S1P-S1P_1_ in the last process, though we cannot exclude a role in adhesion or homing either. Here we demonstrate that S1P_1_ receptor expression is increased in mouse livers in a NASH model, and in previous experiments we have demonstrated that pharmacologic inhibition of this signaling axis with FTY720 ameliorates murine NASH (23). However, the macrophage-specific role of S1P signaling has not been examined in this context. In ongoing experiments we are interrogating the *in vivo* role of myeloid cell S1P_1_ in NASH models, as a corollary to our *in vitro* findings.

## Author Contributions

C-YL designed and conducted experiments, analyzed and graphed data and wrote the manuscript. MS, YG, and AM designed and conducted experiments. AR designed and supervised experiments. HM designed and supervised experiments, analyzed data, and wrote the manuscript. All authors reviewed and approved the manuscript.

### Conflict of Interest Statement

The authors declare that the research was conducted in the absence of any commercial or financial relationships that could be construed as a potential conflict of interest.

## Abbreviations

BMDM: bone marrow-derived macrophages
EVs: extracellular vesicles
FFA: free fatty acids
HPRT: Hypoxanthine-guanine phosphoribosyl transferase
IMH: immortalized mouse hepatocytes
IRE1α: inositol-requiring enzyme 1 α
LCM: L929 cell-conditioned medium
NAFL: Non-alcoholic Fatty Liver
NAFLD: Non-alcoholic Fatty Liver Disease
NASH: Non-alcoholic Steatohepatitis
PA: Palmitic acid
SphK: sphingosine kinase
SphK1: sphingosine kinase 1
SphK2: sphingosine kinase 2
S1P: sphingosine 1-phosphate
S1P_1_: sphingosine 1-phosphate receptor 1.
