# Supplementary material for: Hepatocyte-Derived Lipotoxic Extracellular Vesicle Sphingosine 1-Phosphate Induces Macrophage Chemotaxis
Source: Front Immunol. 2018 Dec 19;9:2980. doi: 10.3389/fimmu.2018.02980 (PMC6305739; doi:10.3389/fimmu.2018.02980)
Supplement: Supplementary file 1 [file Data_Sheet_1.pdf]

Supplementary Material

Hepatocyte-derived Lipotoxic Extracellular Vesicle Sphingosine 1-phosphate Induces Macrophage Chemotaxis

Chieh-Yu Liao[1], Myeong Jun Song[1], Yandong Gao[2], Amy S. Mauer[1], Alexander Revzin[2], Harmeet Malhi[1]*

*Corresponding author: Harmeet Malhi, M.B.B.S: Malhi.harmeet@mayo.edu

1. Supplementary Figure 1
2. Supplementary Figure 2
3. Supplementary Figure 3

Supplementary Figure 1.

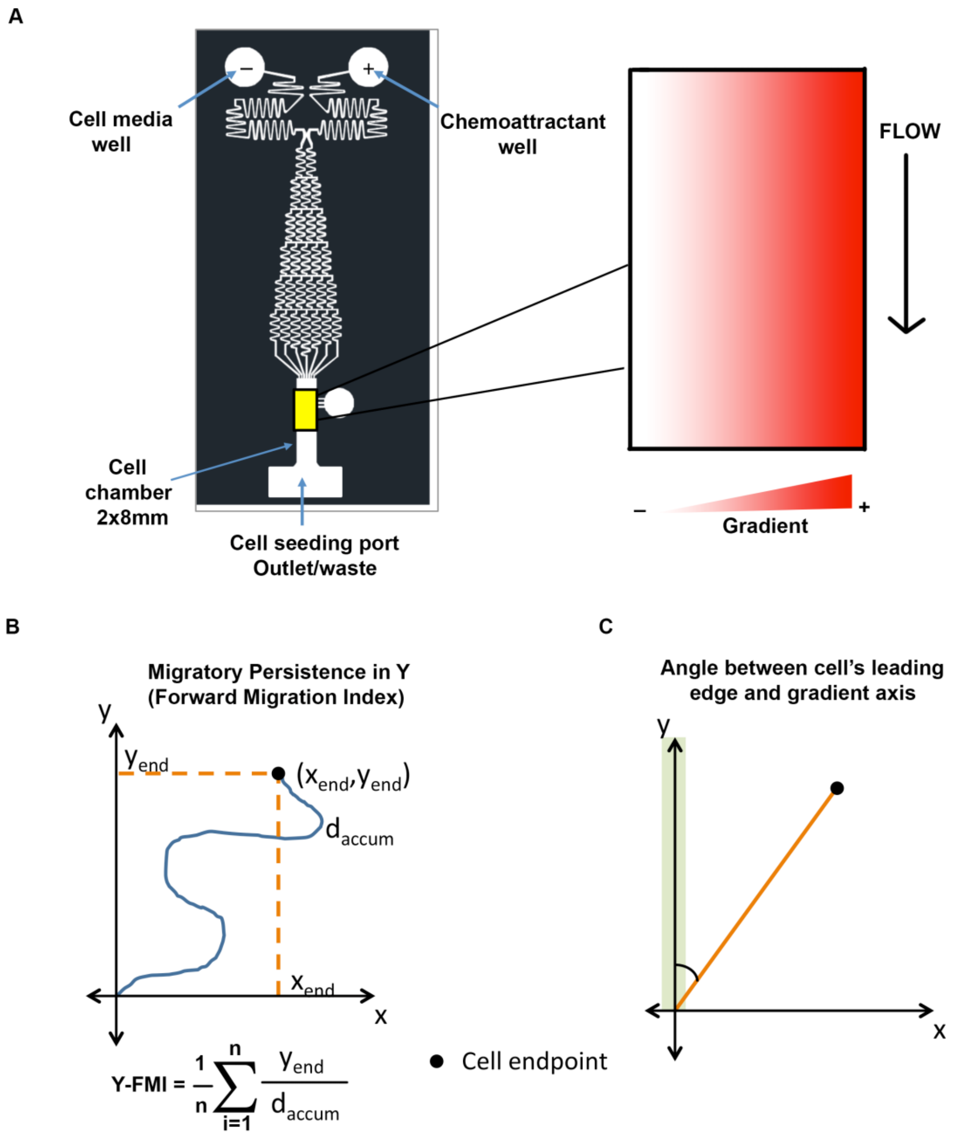

Supplementary Figure 1. The microfluidic gradient generator and calculation of measured values. (A) A schematic representation of the microfluidic gradient generator. Two circular wells on top are for the application of chemoattractant and chemoattractant-free media. The chemoattractant is mixed and equilibrated through the micro-channels in the middle, such that there is a gradient generated in the cell chamber that's perpendicular to the direction of flow. **(B)** Calculation of the forward migration index (migratory persistence in Y). **(C)** Representation of the angle between the cell's leading edge (orange) and gradient axis (green).

Supplementary Figure 2.

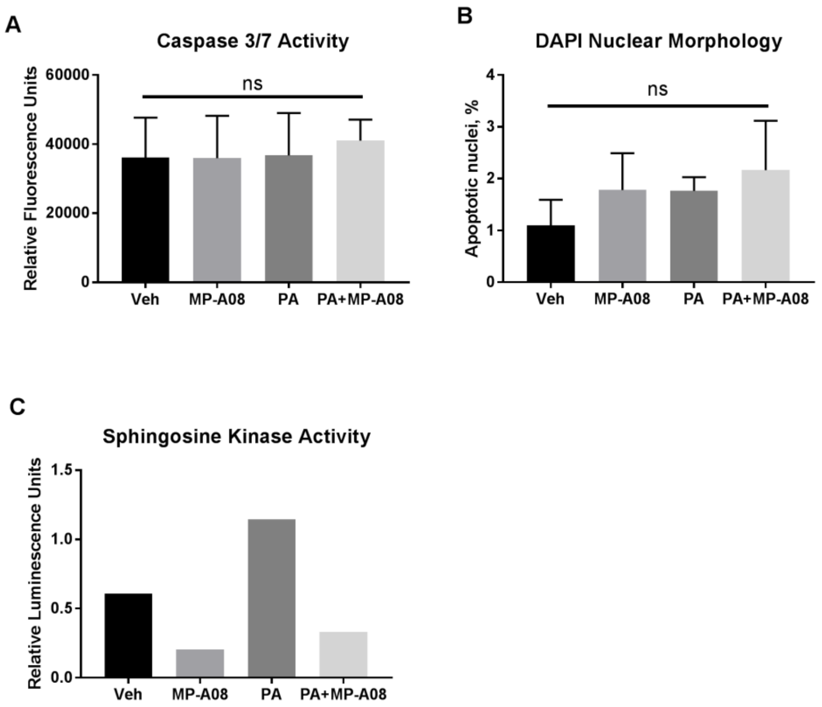

Supplementary Figure 2. Hepatocyte apoptosis is not increased by sphingosine kinases 1 and 2 inhibitor MP-A08. (A) Biochemical assessment of apoptosis by caspase 3/7 activity in IMH cells treated with 400 µM PA, 400 µM PA + 2 µM MP-A08, vehicle, or vehicle + 2 µM MP-A08 for 20 hours. **(B)** Morphologic assessment of apoptosis by assessment of DAPI stained nuclear morphology in IMH cells were treated with 400 µM PA, 400 µM PA + 2 µM MP-A08, vehicle, or vehicle + 2 µM MP-A08 for 20 hours. **(C)** Sphingosine kinase activity in IMH cells were treated with 400 µM PA, 400 µM PA + 2 µM MP-A08, vehicle, or vehicle + 2 µM MP-A08 for 20 hours. Ns=not significant.

Supplementary Figure 3.

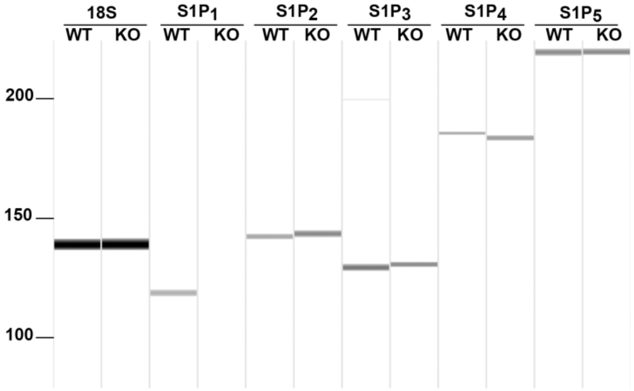

Supplementary Figure 3. mRNA expression of sphingosine 1 phosphate receptors in bone marrow derived macrophages. Capillary electrophoresis was used to resolve PCR products to detect the presence of S1P receptors 1-5 in wildtype (WT) and S1P$_1$ receptor knockout (KO) bone marrow derived macrophages. 18S was used as a control. S1P$_1$ was only detected in WT macrophages. S1P 2-5 mRNA was detected in both WT and KO macrophages.
